# Central autonomic network mediates cardiovascular responses to acute inflammation: Relevance to increased cardiovascular risk in depression?

**DOI:** 10.1016/j.bbi.2013.02.001

**Published:** 2013-07

**Authors:** Neil A. Harrison, Ella Cooper, Valerie Voon, Ken Miles, Hugo D. Critchley

**Affiliations:** a Clinical Imaging Sciences Centre, Brighton and Sussex Medical School, University of Sussex Campus, Falmer, Brighton BN1 3AR, UK; b Sackler Centre for Consciousness Science, University of Sussex, Falmer BN1 9RR, UK; c Sussex Partnership NHS Foundation Trust, Sussex Education Centre, Mill View Hospital, Nevill Road, Hove BN3 7HY, UK; d Department of Psychiatry, Behavioural & Clinical Neurosciences Institute, University of Cambridge, Cambridge CB2 0QQ, UK

**Keywords:** Cytokines, FDG PET, Peripheral inflammation, Blood pressure, Sympathetic, Heart rate variability

## Abstract

Inflammation is a risk factor for both depression and cardiovascular disease. Depressed mood is also a cardiovascular risk factor. To date, research into mechanisms through which inflammation impacts cardiovascular health rarely takes into account central effects on autonomic cardiovascular control, instead emphasizing direct effects of peripheral inflammatory responses on endothelial reactivity and myocardial function. However, brain responses to inflammation engage neural systems for motivational and homeostatic control and are expressed through depressed mood state and changes in autonomic cardiovascular regulation. Here we combined an inflammatory challenge, known to evoke an acute reduction in mood, with neuroimaging to identify the functional brain substrates underlying potentially detrimental changes in autonomic cardiovascular control.

We first demonstrated that alterations in the balance of low to high frequency (LF/HF) changes in heart rate variability (a measure of baroreflex sensitivity) could account for some of the inflammation-evoked changes in diastolic blood pressure, indicating a central (rather than solely local endothelial) origin. Accompanying alterations in regional brain metabolism (measured using ^18^FDG-PET) were analysed to localise central mechanisms of inflammation-induced changes in cardiovascular state: three discrete regions previously implicated in stressor-evoked blood pressure reactivity, the dorsal anterior and posterior cingulate and pons, strongly mediated the relationship between inflammation and blood pressure. Moreover, activity changes within each region predicted the inflammation-induced shift in LF/HF balance. These data are consistent with a centrally-driven component originating within brain areas supporting stressor evoked blood pressure reactivity. Together our findings highlight mechanisms binding psychological and physiological well-being and their perturbation by peripheral inflammation.

## Highlight

► Acute inflammation modulates two cardiovascular risk factors: blood pressure and heart rate variability by effects on brain homeostatic control and integration centres.

## 1 Introduction

Inflammation is a risk factor common to depression and cardiovascular disease and is implicated in the increased co-morbidity observed for both these conditions (Panagiotakos et al., 2004). Patients with major depression show activation of inflammatory pathways, manifest as increases in pro-inflammatory cytokines in both the circulation (Alesci et al., 2005) and cerebrospinal fluid (Levine et al., 1999). Some studies also demonstrate a positive correlation between plasma concentrations and symptom severity (Alesci et al., 2005; Thomas et al., 2005).Inflammation even impacts on discrete depression-related symptoms such as fatigue, insomnia and anger/hostility (Dantzer et al., 2008). These motivational and affective changes can be observed in individuals not meeting full criteria for major depression (Suarez et al., 2002, 2004). The link between inflammation and mood symptoms is apparent across the lifespan (Raison et al., 2006).

There is increasing biological understanding of the relationship between physical and psychological health. Functional polymorphisms within pro-inflammatory cytokine genes such as Interleukin-1beta (IL-1beta) and tumor necrosis factor-alpha (TNF-alpha) may increase the risk of depression and are associated with reduced responsiveness to conventional antidepressant therapy (Yu et al., 2003; Jun et al., 2003). Further, experimental induction of acute inflammation using either typhoid vaccine (Harrison et al., 2009a) or lipopolysaccharide infusion (Reichenberg et al., 2001) results in an acute reduction in mood and the therapeutic use of pro-inflammatory cytokines such as Interferon-alpha or IL-2 in the clinical management of medical patients will induce major depressive episodes in up to 50% of patients (Musselman et al., 2001).

Inflammation is also implicated in the aetiology of cardiovascular disease (Ridker et al., 1997). Two broad patterns of inflammation-associated cardiovascular risk are recognized: First, an association between chronic low-grade inflammation and gradual accumulation of atherosclerosis and second, an association between acute inflammation (e.g. viral infection) and a transiently increased risk of acute cardiovascular events (Hansson, 2005).This is of particular interest given the association between depression and cardiovascular disease, which remains even after controlling for conventional risk factors such as medication, body-mass index, physical activity, hypertension and hypercholesterolemia (Wulsin and Singal, 2001). Thus understanding the interaction between depression and inflammation is important for mitigating cardiovascular risk (Panagiotakos et al., 2004).

Cardiovascular research has recognised the increased cardiovascular morbidity observed following acute inflammatory episodes such as respiratory or urinary tract infections (Smeeth et al., 2004; Meier et al., 1998) and severe illness requiring intensive care (Quartin et al., 1997). Research into the mechanisms underlying this increased risk has emphasized the role of direct inflammatory effects on endothelial reactivity (Hingorani et al., 2000), as has been observed for other conventional risk factors (Kinlay and Ganz, 1997). Supporting this proposal, experimental induction of acute inflammation using Typhim vaccine suppresses bradykinin- and acetylcholine- induced relaxation of arterial blood vessels (Hingorani et al., 2000). Of note, this impairment in endothelial function follows a very mild systemic inflammatory response (associated with a 2- to 3-fold elevation in pro-inflammatory cytokines) and persists for eight hours after inflammatory challenge (Hingorani et al., 2000).

Chronic mild inflammation has also been linked in cross-sectional studies to essential hypertension (Panza et al., 1990), one of the most important risk factors for cardiovascular disease, with family studies suggesting that chronic mild inflammation precedes blood pressure changes (Zizek et al., 2001). Again direct effects of inflammation on endothelium-dependent vascular reactivity have been proposed as the mediating mechanism (Bautista, 2003, Sinisalo et al., 2000). However, subclinical inflammation in healthy middle-aged adults without overt cardiovascular disease is also associated with changes in autonomic tone (a reduction in heart rate variability (Sajadieh et al., 2004)) which itself is a cardiovascular risk factor associated with both depression (Kemp et al., 2010)and risk of coronary heart disease even in physically healthy individuals (Dekker et al., 2000). This suggests that inflammation may mediate the increased risk of cardiovascular disease, not only through direct effects on endothelial reactivity, but also via centrally-mediated effects on autonomic reactivity indexed by heart rate variability.

Heart rate variability (HRV) is a non-invasive index of beat-to-beat changes in heart rate. Overall HRV reflects parasympathetic neural activity interacting with sympathetic influences on the sinus node of the heart and reflects the capacity for parasympathetic inhibition of autonomic arousal. HRV decreases under both physical and emotional stress and increases with rest. High HRVindicates a healthy autonomic nervous system that can respond flexibly to dynamically changing environmental demands while low HRV is frequently a marker of ill-health. Low HRV is an independent risk factor for cardiovascular morbidity and mortality (Tsuji et al., 1996; Dekker et al., 2000) and may precede inflammation-mediated atherosclerosis (Huikuri et al., 1999). Low HRV is also observed in patients with major depressive disorder (Kemp et al., 2010) even without overt cardiovascular pathology though unlike changes in endothelial function (Broadley et al., 2002) has been shown to at least partially reverse following successful treatment (Carney et al., 2000; Narshoni et al., 2001). This state-like association between low HRV and depression is hypothesized to underlie the link between depression and cardiovascular events including sudden cardiac death (Taylor et al., 2010). However, what factors mediate and sustain this reduction in HRV in depression is currently unclear.

Specific brain regions, notably the subgenual cingulate cortex (Drevets et al., 1997) are implicated in the pathogenesis and clinical expression of depression. Inflammation perturbs activity within this region to predict sickness-related changes toward a negative mood (Harrison et al., 2009a). Importantly, there is functional coupling between subgenual cingulate and adjacent ventromedial prefrontal cortices with posterior cingulate cortex within a default mode network implicated in self-directed cognitive processing (Gusnard et al., 2001). Activity within this network is also inversely coupled to dACC activity, both in terms of engagement with external tasks (Raichle et al., 2001) and importantly in effects on cardiovascular physiology (Critchley et al., 2011; Wager et al., 2008) mediated through pons. Increased metabolic activity within dorsal and posterior cingulate and pons also predict response to antidepressant treatment (Mayberg et al., 2000).

Here we investigate the effects of acute inflammatory challenge on blood pressure and sympathetic/parasympathetic balance using a Typhoid vaccine inflammatory challenge previously shown to induce an acute reduction in mood (Harrison et al., 2009a). Central mediators of the relationship between inflammation and change in blood pressure and sympathetic/ parasympathetic balance were investigated using ^18^Fluorodeoxyglucose (FDG) PET neuroimaging before and 4 h after Typhim or placebo (saline) injection. Specifically we test the hypotheses that (1) direct measures of vascular response to inflammation (blood pressure changes) reflect central adjustments to autonomic control apparent as shifts in LF/HF balance (Mancia et al., 1983); (2) inflammation will induce changes in regional brain activity within the hierarchy of homeostatic brain areas linked to the expression of mood changes and motivation state (e.g. insula, cingulate ventromedial prefrontal cortex (Harrison et al., 2009b) (3) a subset of these areas (cingulate, dorsal pons (Gianaros and Sheu, 2009) will mediate inflammation-induced changes in cardiovascular state.

## 2 Materials and methods

### 2.1 Participants

Twenty healthy male participants (mean 24.7 ± 6.8 years) were recruited from advertisements on a local community website. Nineteen were white-Caucasian and 1 black-African. Volunteers were reviewed by a psychiatrist (N.A.H.) and screened for a history of any relevant physical or psychiatric illness. One participant had a history of mild eczema. Four participants rated their general health as excellent, 9 very good and 7 good. No participant rated his or her general health as poor or fair. All were medication free, with no non-steroidal or steroidal inflammatory drug use in the preceding 2 weeks and were non-smokers. Volunteers who had received typhoid vaccine within 3 years or other vaccine within 6 months were excluded. Participants were advised not to consume caffeinated beverages or alcohol, avoid high-fat meals and refrain from excessive exercise for 24 h prior to testing. All were fasted for a minimum of eight hours prior to testing and consumed only water until completion of the study. After complete description of the study to the participants, written informed consent was obtained. Procedures were approved by the Brighton East National Research Ethics Committee.

### 2.2 Study design

We adopted a randomized, double-blind, repeated measures design in which all participants underwent ^18^fluorodeoxyglucose positron emission tomography (^18^FDG-PET) imaging sessions at baseline then again at 4 h. At the beginning of the study a small calibre venous cannula was inserted into the back of the left hand from which blood samples were taken then ^18^FDG PET tracer administered prior to each scan. PET scanning was performed for 35 min following a 30-min uptake phase. During and one hour prior to each PET scan participants were instructed to lay still with their eyes open in a dimly-lit room. During scanning heart rate was continuously recorded using a Pulse Oxymeter (Nonin 8600 FO Series, Nonin Medical Inc.). Following the first scanning session participants randomly received injections of either 0.025 mg Salmonella-typhi capsular polysaccharide vaccine (Typhim Vi, Aventis Pasteur MSD) or0.5 ml normal saline (placebo) intramuscularly into the deltoid muscle. At the end of the study a high-resolution inversion-recovery echoplanar image was obtained to aid registration of the PET images. Participants also performed memory testing after each PET scan and an additional PET scan 4 h after completion of this study – details of which are not reported here.

Inflammatory Model: We used a Salmonella-typhi vaccination inflammatory model that has previously been shown to induce a low-grade inflammatory response (indexed by a 2- to 3-fold increase in circulating inflammatory cytokines from 2 h after vaccination) and increase in diastolic blood pressure (Harrison et al., 2009b) without associated change in body temperature (Hingorani et al., 2000). There were no complications of either Typhoid or saline injection. Blood (10 ml) was drawn into vacutainer tubes containing EDTA anti-coagulant, centrifuged immediately at 1250*g* for 10 min at room temperature. Plasma was removed, aliquoted and immediately frozen in liquid nitrogen before transfer to a −80 °C freezer for storage. Plasma interleukin-6 (IL-6), interleukin-1 Receptor antagonist (IL-1Ra) and tumor necrosis factor alpha (TNFα) were assessed using high sensitivity ELISAs (R&D Systems, Abingdon, UK). The limit of detection of the IL-6 assay is0.039 pg/mL, with intra- and inter-assay coefficients of variation (CVs) of 7.4% and 7.8%. The IL-1Ra assay had a limit of detection of 6.26 pg/mL and inter- and intra-assay CVs of 5.3% and 8.6% respectively. The TNFα assay had a detection limit of 0.038 pg/mL with intra- and inter-assay CVs of 5.3% and 8.4% respectively. Cytokine analyses were performed using mixed measures ANOVAs in SPSS 16.0.

### 2.3 Physiological data acquisition and analysis

Blood pressure was recorded prior to each scanning session using an electronic blood pressure monitor (Omron M6, Kyoto, Japan). Blood pressure was recorded twice at each session and the mean pressure recorded. Continuous heart rate was recorded throughout each PET scanning session using a pulse oxymeter (Nonin 8600 FO Series, Nonin Medical Inc.). Data were recorded at 500 Hz in Spike 2 version 7 via a Power 1401 Amplifier (Cambridge Electronic Design, Cambridge, UK).Heart rate data were visually inspected, artefact removed and interbeat intervals calculated using custom software in Matlab 2008. Heart rate variability (HRV) within high (0.15–0.4 Hz), low (0.04–0.15 Hz) and very low (0–0.04 Hz) frequency bands were then assessed by calculating the power spectral density (PSD) derived from artefact free 20 min pulse oximetry recording periods. Measures of heart rate variability derived from pulse oximetry has been validated against (less convenient) electrocardiographic methods (e.g. Lu et al., 2008). Lowfrequency/High frequency (LF/HF) heart rate variability ratio was then determined (Berntson et al., 1993; ESC/NASPE Task Force, 1996). Spectral density estimates were calculated in HRV analysis tool version 2.0 (http://bsamig.uku.fi/, Biosignal Analysis and Medical Imaging Group, Department of Physics, University of Kuopio, Finland) using fast fourier transform (FFT) based on Welch’s periodogram method.

All cytokine and physiological data were analysed using mixed measures ANOVA with time as within subject and group as between subject factors in SPSS 19. Regression analyses followed by Goodman test for mediation were used to investigate relationships between inflammatory challenge, change in blood pressure and change in sympathetic/ parasympathetic balance. Mediation analyses were performed using the interactive calculation tool for mediation tests: http://quantpsy.org/sobel/sobel.htm using the Goodman test equation Z score = a*b/SQRT(b2*sa2 + a2*sb – sa2*sb2) where a is the raw (unstandardized) regression coefficient for the association between the independent variable (inflammation) and the mediator (LF/HF), s_a_ is the standard error of a, b is the raw coefficient for the association between the mediator (LF/HF ratio) and the dependent variable (dBP) (when inflammation is also included as a predictor of dBP) and s_b_ is the standard error of b.

### 2.4 Image acquisition and analysis

PET scans were acquired for 35 min starting 30 min after ^18^FDG tracer administration (mean 155.3 ± 11.8 MBq) on a Siemens Biograph 64 PET-CT scanner in 3D dynamic list acquisition mode. Participants lay supine with their eyes open and mild head restraint was applied. Prior to each PET acquisition a low dose CT scan (120 kVp, 10 mA, 0.059 mSv whole body equivalent dose) was acquired for attenuation correction. After correction for scatter, randoms and effects of attenuation, images were reconstructed in 35 one-minute windows using Siemens proprietary iterative 3D reconstruction schema with 21 iterations and 8 subsets. Individual 1 min scans were then realigned and summed to produce a single 35 min activation scan per session which was co-registered to subjects’ structural MRI scans. Activation images were then normalised using the flow fields acquired from segmentation of corresponding structural MRI scans then spatially smoothed with a 12 mm FWHM Gaussian kernel using standard SPM methods.

Activation images were included in subject specific paired samples t-tests using a grand mean scaled value of 50 ml/dl/min and proportional scaling to produce subject specific contrast images (4 h > baseline) corrected for overall cerebral glucose metabolism. These contrast maps were then included in a single level whole brain mediation analysis (Wager et al., 2008) to investigate brain regions mediating the relationship between inflammatory challenge and increase in diastolic blood pressure. Bilateral dorsal Pons and dorsal anterior cingulate were defined as a priori regions of interest for mediating this relationship based on data from an earlier fMRI study (Harrison et al., 2009b) and review of neuroimaging studies of stressor evoked blood pressure reactivity (Gianaros and Sheu, 2009). Regression analyses performed within a general linear model with dummy variable coding of group membership (inflammation, placebo) were then used to investigate whether regions found to mediate the relationship between inflammation and blood pressure change additionally correlated with the interaction between inflammation and change in LF/HF balance.

## 3 Results

### 3.1 Cytokine analyses

**Fig. 1:**
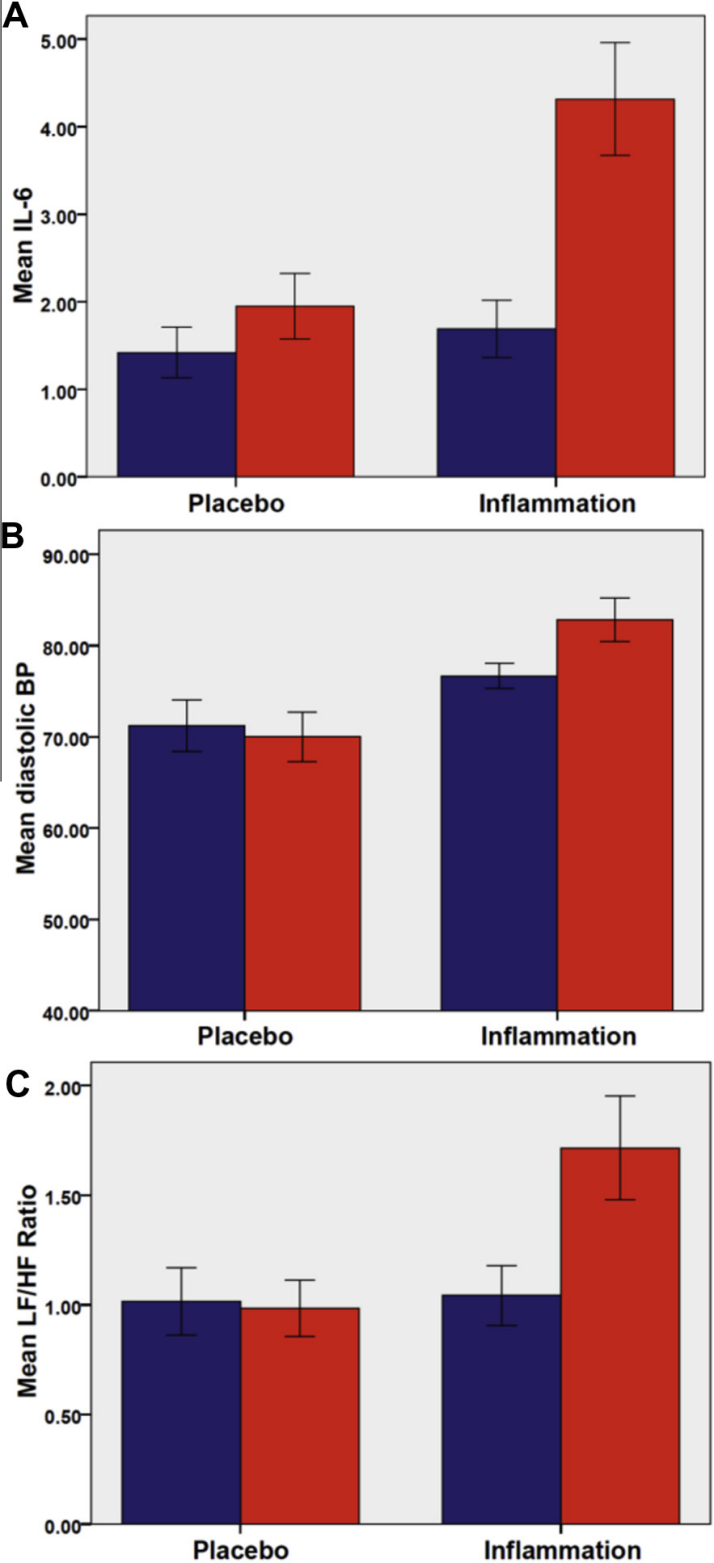
Change in: plasma interleukin-6 (IL-6) levels (A), diastolic blood pressure (B) and LF/HF ratio (C) at baseline (blue) and 4 h (red) after typhim or placebo (saline) i.m. injection. All error bars denote standard error of the mean (s.e.m.). (For interpretation of the references to colour in this figure legend, the reader is referred to the web version of this article.)

Cytokine analysis confirmed a significant increase in systemic inflammation following inflammatory challenge demonstrated by significant group × time interactions for both IL-6 (*F*_(1,18)_ = 6.91, *p* = 0.017) (Fig. 1 A) and IL-1Ra (*F*_(1,18)_ = 11.77, *p* = 0.003). Main effects of group and time were non-significant at *p* < 0.05. As observed previously using the typhoid vaccine inflammatory model (Brydon et al., 2008), TNF-alpha levels were not significantly altered (Group × time interaction: *F*_(1,18)_ = 2.49, p = n.s.).

### 3.2 Cardiovascular responses

Inflammation but not placebo was associated with a significant increase in diastolic blood pressure (Group × time interaction: *F*_(1,18)_ = 5.88, *p* = 0.026),main effect of time *F*_(1,18)_ = 2.65, *p* = n.s. (Fig. 1B). Systolic blood pressure was unchanged (Group × time interaction: *F*_(1,18)_ = 0.07, *p* = n.s). LF/HF ratio significantly increased across the duration of the study (Main effect of time: *F*_(1,18)_ = 6.72, *p* = 0.02) likely associated with the psychological stress of being enrolled in the study with a significantly greater increase following inflammation versus placebo (Group × time interaction: *F*_(1,18)_ = 8.06, *p* = 0.01, Fig. 1C). Exploration of this change in LF/HF ratio following inflammation showed that the effect was mediated predominantly by an increase in low frequency cardiac influences (Group × time interaction: *F*_(1,18)_ = 4.32, *p* = 0.05) with a smaller(non-significant) reduction in high frequency (parasympathetic) components observed (Group × time interaction: *F*_(1,18)_ = 1.80, *p* = 0.19).

**Fig. 2:**
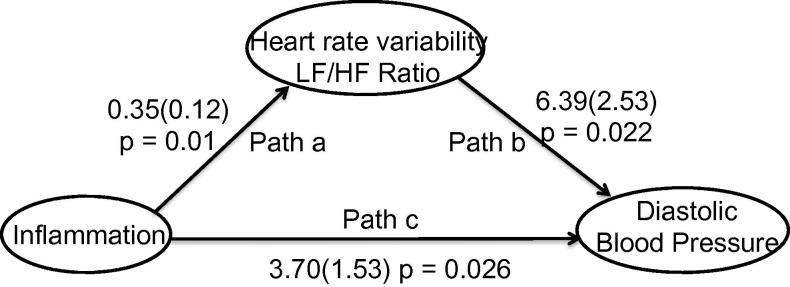
Mediation of effects of inflammatory challenge on diastolic blood pressure change by change in LF/HF ratio. Path c denotes direct relationship between inflammation and blood pressure change. Path a the relationship between LF/HF and inflammatory challenge and path b the relationship between diastolic blood pressure and inflammatory challenge mediated by change in LF/HF ratio.

Bivariate correlations of inflammatory status (inflammation/ placebo group), change in diastolic blood pressure and change in LF/HF ratio demonstrated significant correlations between each factor: Group and BP change (Pearson = 0.53 *p* = 0.017), Group and change in LF/HF ratio (Pearson = 0.56 *p* = 0.01), change in LF/HF ratio and BP (Pearson = 0.66, *p* = 0.002). Subsequent mediation analysis of these bivariate relationships demonstrated that the relationship between inflammation and change in blood pressure was mediated by central effects on LF/HF ratio (Goodman Test = 1.97, *p* = 0.049, Fig. 2).

### 3.3 Neural mediation of inflammatory effects on diastolic blood pressure

**Fig. 3:**
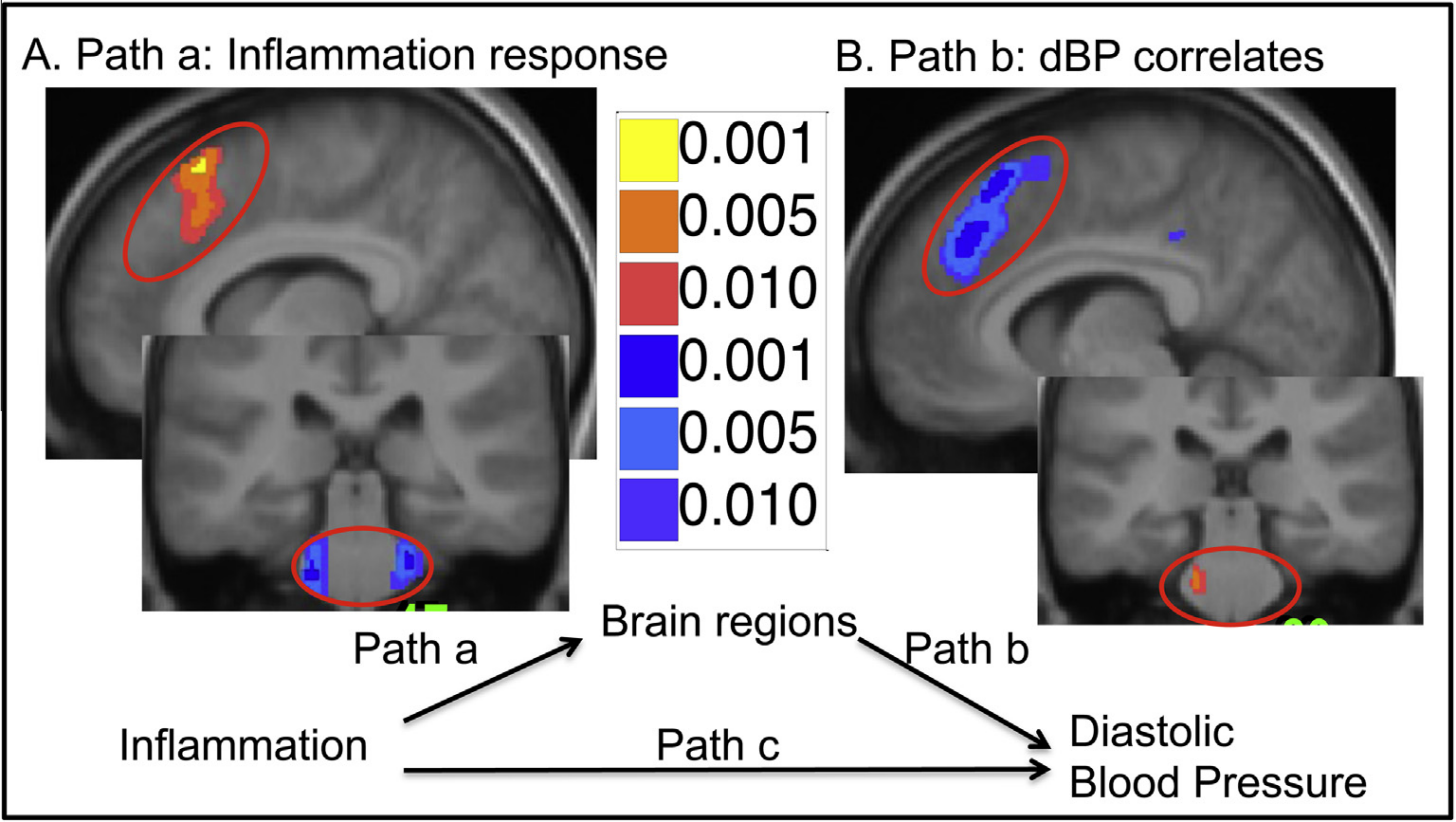
Results of whole brain mediation analysis, red-yellow denote positive correlations and blue-purple negative. (A) Brain regions correlating with a change in inflammatory status showing a significant increase in resting FDG uptake in the anterior cingulate and decrease in bilateral pons. (B) Brain regions correlating with change in diastolic blood pressure showing decrease in resting FDG uptake in the anterior cingulate and increase in left pons.

**Fig. 4:**
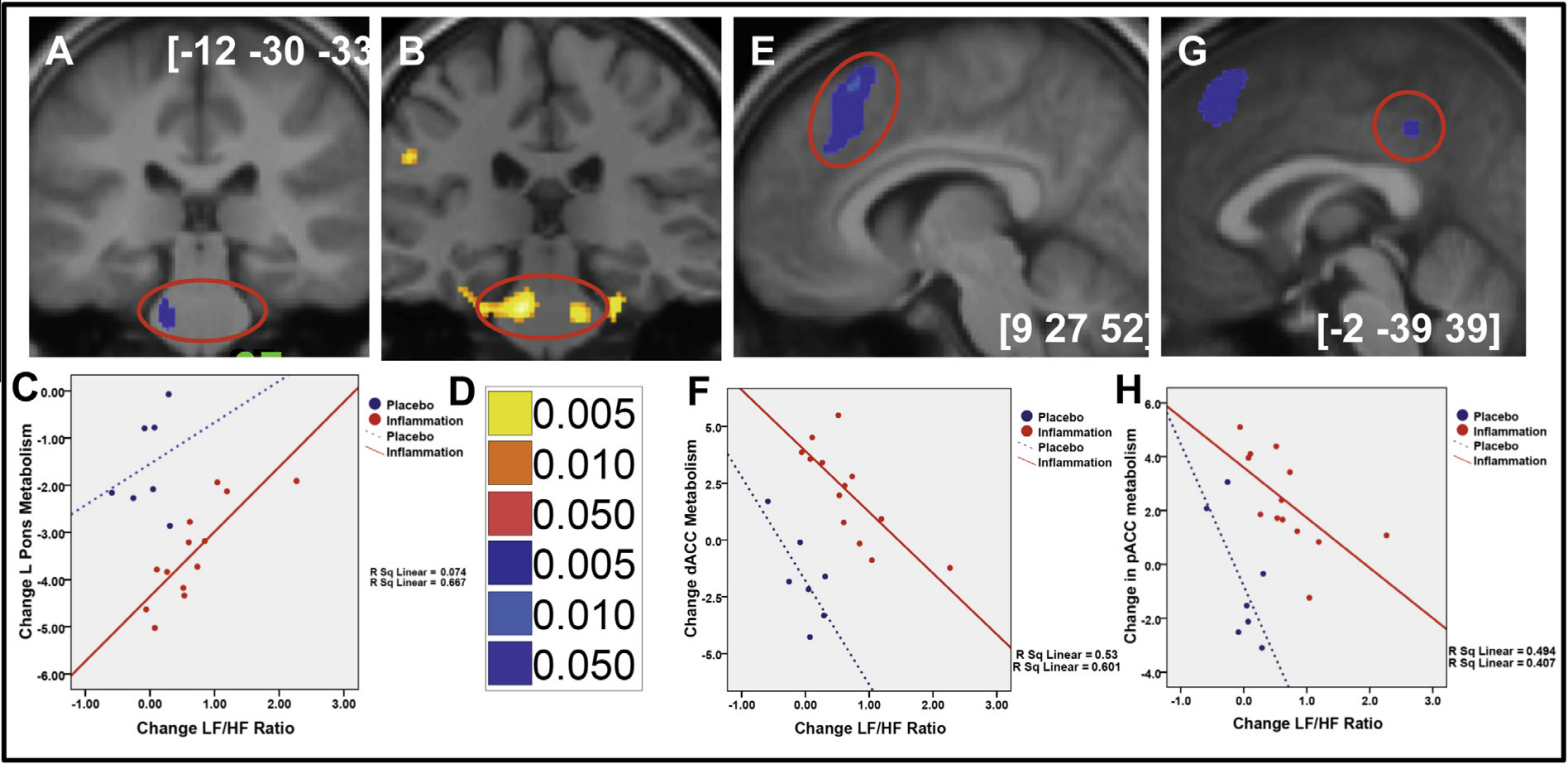
Results of whole brain mediation analysis illustrating regions showing a significant mediation of effects of inflammation on blood pressure by change in LF/HF ratio. (A) Left pontine region showing a significant negative mediation by LF/HF ratio change. (B) Location of bilateral pontine correlations with Typhim induced mean arterial pressure change in an earlier independent fMRI study (from Harrison et al. 2009b). (C) Correlation between left pontine FDG uptake and LF/HF change, demonstrating significant reduction in activity following inflammation and interaction between inflammation and change in LF/HF ratio. (D) Statistical threshold for mediation analysis data. (E) dACC region showing a significant negative mediation by LF/HF ratio change. (F) Correlation between dACC FDG uptake and LF/HF change, demonstrating significant increase in activity following inflammation and interaction between inflammation and change in LF/HF ratio. (G) pCC region showing a significant negative mediation by LF/HF ratio change. (H) Correlation between pCC FDG uptake and LF/HF change, demonstrating significant increase in activity following inflammation and interaction between inflammation and change in LF/HF ratio.

Whole brain mediation analysis was next performed using our FDG-PET imaging data to identify the neural mediators of inflammatory effects on diastolic blood pressure. Mediation analysis firstly confirmed the previously described matrix of brain regions including Insula, dorsal anterior cingulate and dorsal pons in which resting glucose metabolism was sensitive to inflammatory challenge (path a in Fig. 3).A subset of these regions including dorsal anterior cingulate cortex and pons also correlated with inflammatory effects on diastolic blood pressure (path b in Fig. 3) suggesting a role in calibrating cardiovascular reactions to meet the adaptive behavioural and metabolic demands of acute inflammatory challenge. Three regions: Right (extending across the midline) dorsal ACC, and left dorsal pons (both *a-priori* regions of interest) together with left posterior ACC additionally survived formal mediation analysis (Table 1, path ab, Fig. 4) suggesting a specific role in mediating the effects of inflammation on diastolic blood pressure.

To determine whether these three regions were also sensitive to effects of inflammation on autonomic balance we performed regression analyses (within a GLM with inclusion of a dummy variable to code inflammation/placebo) on each region (Fig. 4C, F, H). This revealed that each of the three regions mediating the relationship between inflammation and change in diastolic blood pressure additionally correlated with change in LF/HF ratio. Left pons, main effect of group (*F*_(1,16)_ = 39.30, *p* < 0.0001), group × change in LF/HF ratio interaction (*F*_(1,16)_ = 7.08, *p* = 0.006), dorsal ACC main effect of group (*F*_(1,18)_ = 49.95, *p* < 0.0001), group × change in LF/HF ratio interaction (*F*_(1,16)_ = 11.00, *p* = 0.001), posterior cingulate cortex main effect of group (*F*_(1,16)_ = 24.29, *p* < 0.0001), group × change in LF/HF ratio interaction (*F*_(1,18)_ = 6.50, *p* = 0.009). This suggests that each region contributes to inflammatory effects on resting blood pressure via a shift in autonomic balance away from cardio protective LF/HF balance.

## 4 Discussion

First, we show that acute inflammatory challenge using Typhim vaccination is associated with a significant increase in diastolic blood pressure at four hours. This finding replicates our previous data using this experimental model (Harrison et al., 2009b). We then show that Typhoid vaccination is also associated with an acute increase in the ratio of low to high frequency changes in heart rate variability (LF/HF) driven predominantly by an increase in low frequency components. LF/HF ratio (and LF power) is believed to reflect baroreflex modulation of cardiovascular autonomic outflow (Goldstein et al., 2011) and is a known risk factor for cardiovascular morbidity and mortality (Dekker et al., 2000). Previous studies investigating cardiovascular effects of acute inflammation using this experimental model (Hingorani et al., 2000) demonstrate a marked reduction in endothelial reactivity to endothelium-dependent vasodilators (e.g. bradykinin and acetylcholine) suggesting that acute inflammation may mediate an increased cardiovascular risk via direct endothelial effects. However, blood pressure and heart rate responses to vasoconstrictors such as endothelin, angiotensin II and phenylephedrine in mice are generally not modulated by chronic IL-6 infusion (Boesen and Pollock, 2007). Our current data implicate an additional centrally mediated mechanism through which changes in diastolic blood pressure (predominantly governed by sympathetic vascular tone) are made in response to immune-brain signalling of inflammatory status possibly by modulation of the baroreflex. They extend the local (endothelial) model of inflammatory effects on cardiovascular risk to encompass centrally regulated autonomic mechanisms.

Our interpretation is supported by our mediation analysis that showed that the effects of inflammation on resting diastolic blood pressure were mediated via a shift in LF/HF ratio. An increase in baroreflex sensitivity (as suggested by the observed increase in LF/HF ratio might be expected to be accompanied by a reduction in heart rate, though within our data this was not observed. Our FDG-PET brain imaging data extends this conclusion by demonstrating that three discrete regions previously implicated in stressor evoked blood pressure reactivity namely dorsal anterior cingulate, posterior cingulate cortex and dorsal pons were sensitive both to inflammatory status and associated change in diastolic blood pressure. Moreover, each region fulfilled formal mediation criteria and was sensitive to the interaction between inflammatory status and change in LF/HF ratio. This suggests a direct role in mediating inflammatory effects on blood pressure via modulation of baroreflex sensitivity. Interestingly, IL-6 has also been implicated in the pathogenesis of hypertensive responses to acute stress with IL-6 knockout mice showing a blunting of hypertensive responses to stress despite a lack of change in heart rate, plasma norepinephrine or phenylephedrine-induced vasoconstrictor responses (Lee et al., 2004). These rodent data suggest a dependency of blood pressure responses to stress on IL-6, not via effects on alpha-1 adrenergic responsiveness but instead by modulation of the effects of these systems on vascular tone, perhaps mediated by changes in baroreflex responsivity.

Animal studies using stimulation, lesion or functional anatomic techniques have identified a circumscribed network of interacting cortical, subcortical and brainstem structures, the central autonomic network, that act to integrate autonomic cardiovascular responses with changing behavioural and metabolic demands (Cechetto and Saper, 1990). Activity within central autonomic nuclei within the brainstem, particularly the pontine raphe and locus ceruleus nuclei ultimately regulate sympathetic and parasympathetic outflow to the body. However, integration of autonomic outflow with contextually adaptive behaviour is supported by bidirectional projections to discrete cortico-limbic structures including anterior cingulate, ventromedial prefrontal cortex, insula and amygdala that underpin emotional and volitional behaviours (Critchley et al., 2011). Functional neuroimaging studies of behaviorally coupled cardiovascular challenges reveal a similar functional architecture in humans. For example, sympathetically mediated blood pressure changes to cold pressor and isometric exercise tasks correlate with activity changes in medial and orbital prefrontal cortex, dorsal anterior cingulate, medial and lateral thalamus, midbrain and pons demonstrating a common architecture underpinning integrated cardiovascular responses to diverse physiological stressors (Critchley et al., 2000; Harper et al., 2000).

A similar network of structures, notably perigenual (pACC) and dorsal anterior cingulate (dACC), insula, amygdala and pons with the addition of the posterior cingulate cortex (pCC), hasalso been implicated in regulating blood pressure changes to a range of cognitive stressors (Gianaros and Sheu, 2009). Activity within dACC correlates with sympathetic arousal during performance of the cognitive Stroop task (Critchley et al., 2005), is engaged by pain-related anxiety (Vogt et al., 2003) and is engaged during the intentional regulation of sympathetic electrodermal response (Critchley et al., 2002). We have also previously demonstrated increases in dACC reactivity to inflammatory stress following Typhim vaccine and awareness of associated fatigue (Harrison et al., 2009b). Together these studies point to the dACC as a mediator of changes in sympathetic arousal supporting volitional, cognitive and emotional behaviours. Connectivity of the pons to cortico-limbic components of the central autonomic network, notably the amygdala, is also believed to be instrumental for interrelating cortical processes supporting stressor-evoked changes in behaviour and cardiovascular reactivity (Gianaros and Sheu, 2009).

It is therefore notable that two key components of the central autonomic control network, dACC and pons show significant mediation of the inflammatory effect on blood pressure and autonomic balance. Further these results provide the first independent replication of our earlier reported findings (using fMRI) of dACC sensitivity to inflammatory status and pons to inflammation-induced changes in mean arterial pressure. Interestingly, in our previous study (Harrison et al., 2009b) though we showed a positive correlation between pontine reactivity and inflammatory effects on blood pressure we failed to demonstrate an increase in reactivity to inflammation per se. This may perhaps be explained by our current data that shows a *reduction* in glucose uptake within the pons following inflammation (Fig. 3A). Similarly with the dACC activity, while previously we demonstrated an increase in reactivity to inflammatory challenge (as replicated in our current data Fig. 3A), no *positive* correlation with blood pressure was observed. Importantly, though we interpret these regions as mediating inflammatory effects on blood pressure, mediation analyses cannot definitively determine causality i.e. it is also plausible that changes in blood pressure mediate inflammation associated changes in dACC activity (perhaps representing altered afferent traffic from baroreceptors reflected in HRV changes). Indeed, mediation analysis of this alternate causal path remains significant, albeit at a lower statistical threshold, suggesting contributions from both pathways. Dual influences from these ascending and descending pathways may also account for our somewhat surprising finding of a negative correlation between dACC activity and change in blood pressure and LF/HF ratio (Fig. 4E and F). Speculatively, this decrease in dACC may reflect relative contributions of top-down prediction and bottom-up afferent information flow in the representation and control of internal bodily state i.e. in an inflamed state, dACC expresses a low signal when a rise in blood pressure (associated with increased afferent traffic from baro receptors) is predicted and a high signal when blood pressure changes are not predicted. Correspondingly, the pons shows the proximate relationship with peripheral state notably diastolic blood pressure and, in the context of inflammation, dACC activity appears to be effecting a regulatory influence on brainstem visceromotor control. A similar dissociated role for these interconnected visceral control centres is also observed in other contexts (e.g. in peripheral autonomic denervation Critchley et al., 2000, 2001).

The role of the pCC in mediating effects of inflammation on diastolic blood pressure is also worthy of comment. Though the pCC is not formally part of the central autonomic network and indeed shows few (if any) direct projections to brain stem pre-autonomic and cardiovascular regulatory cell groups, several studies have shown changes in pCC activity in conjunction with stressor evoked autonomic and cardiovascular reactions (Gianaros and Sheu, 2009). This apparent discrepancy has recently been interpreted in the context of the default mode literature where the pCC is proposed tofoster inwardly directed attention to interoceptive (e.g. autonomic) information (Raichle et al., 2001) and support self-relevant evaluative processes including the automatic appraisal of unpleasant self-relevant stimuli (Maddock, 1999). In particular, one study (Wong et al., 2007) showed that though pCC activity decreases as exercise induced changes in heart rate increase, the temporal association of these changes is weaker than observed in other visceromotor cortices. Wong thus proposed that pCC activity changes may relate more to processes associated with evaluative appraisal of self-relevant stressors and only indirectly (or perhaps even spuriously) to associated changes in autonomic and cardiovascular functioning. It is therefore possible that the changes in pCC activity observed in our current study also relate to associated changes in evaluative appraisal of the inflammatory challenge rather than changes in autonomic control per se.

To conclude, to date explanations for why HRV may be reduced in depression have typically adopted cognitive explanations for example, an inability to disengage threat detection that then serves to perpetuate worry and hyper-vigilance, even when no real threat exists (Thayer and Lane, 2000). However our current data suggests an alternate physiological interpretation. Namely, that depression associated increases in LF/HF ratio result from both direct and indirect effects of cytokines and inflammation on the central autonomic network. The present study (which reports responses to inflammation in a healthy non-depressed population) does not permit us to directly test this alternate hypothesis. Nevertheless, our data provide an insight that warrants further investigation in future studies on cardiovascular and autonomic reactivity in a depressed population. Together our study provides data supporting a novel alternate explanation for the reduced HRV and associated increased cardiovascular risk observed in patients with depression. Importantly, recent data suggests clinical efficacy of anti-inflammatory therapies in depressed patients (Tyring et al., 2006). Our data suggests that anti-inflammatory therapies may also serve to correct potentially pathological changes in heart rate variability mitigating the higher cardiovascular risk observed in this vulnerable population. This is particularly relevant given that impairments in endothelium-dependent flow mediated dilatation has been shown to remain impaired even in remitted patients following conventional anti-depressant therapies (Broadley et al., 2002).

The present study is constrained in the degree to which we can extrapolate the findings linking sickness responses and bodily inflammation to changes in mood and autonomic states reported in primary idiopathic depression. Firstly, the observed autonomic responses to Typhoid vaccination do not completely replicate the pattern of cardiac parasympathetic withdrawal most commonly reported in depression (Kemp et al., 2010) and other forms of psychopathology associated with anxiety and rumination (Baer et al., 2007; Ottaviani et al., 2009). Moreover, studies of cytokines in idiopathic depression typically require large group sizes to demonstrate significant associations, consistent with mild cytokine elevation in the majority of depressed patients and the associated ‘acute on chronic’ nature of cardiovascular risk. This contrasts with the greater cytokine elevations associated with vaccination and interferon treatment. Arguably, the visceral afferent effects of inflammation on mood and autonomic responses are more closely linked to psychomotor retardation and related negative depressive symptoms (Brydon et al., 2008) rather than anxiety and agitation that occur co-morbidly in over half of depressed patients. Similarly, our use of inflammation as a model for understanding cytokine effects on mood and brain mediators of autonomic effects may apply most strongly to a subcategory of patients with primary depression e.g. melancholia as well as to those with some forms of secondary depression. Ultimately, we need enhanced understanding, with greater sensitivity across method, to strengthen the application of psychoneuroimmunology for personalized management of primary depression. This study generates a number of useful questions toward this broader aim.

## Figures and Tables

**Table 1:** Brain regions mediating relationship between inflammation and diastolic blood pressure (path ab).

Side	Region	Coordinates	Peak Z score	Cluster	Peak *p* value
*L*	Dorsal Pons	[−12–30 −33]	2.41	109	0.016
*R*	Dorsal ACC	[9 27 52]	2.77	1215	0.0056
*L*	Posterior ACC	[−2–39 39]	2.07	67	0.038
